# Two Recent Cases of Excision of the Vermiform Appendix for Chronic Relapsing Appendicitis in the Intervals

**Published:** 1894-01

**Authors:** Thomas G. Morton

**Affiliations:** Senior Surgeon Pennsylvania Hospital, Philadelphia; Philadelphia, Pa.


### TWO RECENT CASES OF EXCISION OF THE VERMIFORM APPENDIX FOR CHRONIC RELAPSING APPENDICITIS IN THE INTERVAL.

---

BY THOMAS G. MORTON, M. D.,
Senior Surgeon Pennsylvania Hospital, Philadelphia.
PHILADELPHIA, PA.

Joseph H. H., aged twenty-four, has been suffering for eighteen months with occasional attacks of severe pain in the right ileo-cæcal region, with the usual symptoms attending catarrhal appendicitis; the first attack, in the spring of 1891, was a severe one; he was unable to attend to his business for some six weeks: the second lasted only a few days, and subsequently he had four attacks, each more or less marked; the last he had before coming to Philadelphia was particularly severe, indeed very threatening, severe pain, not only local but extending to the bladder, circumscribed swelling and hardness, constipation, irregular chills and fever; recovery protracted

The patient's condition during the attack was indeed so alarming that it was deemed best he should seek operative relief. Examination disclosed tenderness on pressure and moderate hardness in the appendix region, and with the history an operation was advised, and two days subsequently a lateral section was made; the appendix was found firmly bound down, as was the case with the caecum, by adhesions; the organ was tied off as was its mesentry; it measured rather more than three inches in length; it was greatly distended near its middle, and the proximal and distal ends were thickened and swollen; a section showed total obstruction of the organ except its middle or distended portion, which was filled with about two drachms of very offensive pus.

No unfavorable symptoms followed, and the patient returned to his home in Clearfield County, on the fourteenth day afterward, and has been quite well since. It is probable that the thinned pus sac would soon have ruptured into the abdominal cavity.

Mr. G. H., aged thirty-four, of Huntingdon, W. Va., has written as follows:

My first attack, on April 25, 1892, was a severe one; sharp pains in the bowels, intestines swelled and became very hard; felt knotted; continuous vomiting. Relief obtained only through copious rectal injections and bowels moved on third day; able to be out, though very weak, in ten days.

The second attacked occurred about the beginning of June, 1892; pains severe, symptoms same as in first attack, though not so intense. Cathartics acted, in conjunction with injections, after about eighteen hours; out in five days.

Next attack was in September; same symptoms as in other attacks, not so severe, however, as the former two. Cathartics gave relief without injection; out in four days.

The fourth attack was in December, was severe, in bed a week; cathartics and injections both used; pain worse than in other attacks.

The fifth attack was in April, 1893; the attack was slight, lasting only three days; same symptoms, no injection used, only salts as a cathartic.

The sixth attack was in July, and the symptoms were mild.

Last attack commenced September 20; was seized with severe pains in the middle of the night; no relief of bowels for twenty-four hours; semi-unconsciousness at intervals, but whether from the pain or effects of morphia cannot say. Copious vomiting; injections only used; stomach much distended and very hard. Relief came on second day.

In all the attacks morphia was used to ease the pain, and Rochelle and Epsom salts were the cathartics employed.

Early in September Mr. H. reached Philadelphia, much broken down by the series of attacks, especially the one just recovered from; and feeling satisfied that he could not live through another seizure, and there being no question of the appendix being the source of the trouble, a lateral section was made October 6th; on lifting up the cæcum the appendix was found enormously elongated, or stretched, measuring probably seven inches, its distal end was attached far up to the side of the ascending colon; when this attachment was separated the organ contracted to about four inches; it had a continuous mesentery its entire length, which when tied, the organ was excised.

Convalescence and final recovery was slow; after the operation there was considerable pain, no special temperature, but more or less colic, and great trouble was experienced in getting the bowels to act, calomel was at first given in minute doses, this was followed by salts, and the bowels inaction continued for rather more than two days, when relief followed injections of glycerin; more or less pain at times seemed to indicate that the intestines had not regained their normal position, but gradually the symptoms subsided, but not until the fourth week did the bowels act without injection. The suture points and the surroundings of the abdominal wound for the first few days were more or less inflamed and irritable, but no further trouble was experienced; the distension of the abdomen from flatus probably brought about wound and suture tension and the slight surrounding inflammation.

Mr. H. was about the house at the end of the fourth week, and seems quite well, the bowels acting without assistance.

"*Examination of the appendix made by Dr. W. K. Walker, of the Orthopædic Hospital.* The tube which is 3⅛ inches long, is firm and rigid so that it can be held by one extremity in the horizontal position without bending in the slightest degree. The vessels of the peritoneal coat are slightly injectcd.

"The lumen of the tube is narrowed, mucous membrane thickened and found covered throughout with a tenacious mucous.

"Near the distal end of the appendix is found a hardened mass of inspissated mucous and fæces one inch in length and one-sixteenth of an inch in diameter.

"The mucous membrane is everywhere injected, but most markedly at the distal extremity of the tube (for a space of 1¼ inches) in which are seen several punctiform hemorrhages with two of considerable size (about $\frac{1}{16}$ by ¼ inch).

"At the point of location of the plug of inspissated mucous and feces are seen two very small necrotic areas (about the size of a pin's head) involving only the mucous membrane, and one of the same size and character, one-half inch from the proximal end of the tube.

"*Microscopical Examination.* A specimen of the mucous placed under the microscope shows an abundance of pus cells with some red blood-corpuscles.

"A section of the tube shows thickening of all the coats, but particularly the mucous, enlarged bloodvessels, and in the hemorrhagic areas, infiltration with red blood-corpuscles."

Of the death rate after operation for appendix, excission in the interval between attacks we have no definite information, but recently (October 29th), Dr. W. T. Bull has written, asking for the number of such cases I have operated upon, the number of deaths, and the cause of death ; so that we may soon expect to have an estimate of the mortality in this operation; I was only able to furnish Dr. Bull with three cases, all of which recovered ; but I am disposed now to consider the operation, a more serious one than formerly, for in each instance in the cases I have had under care, unusual difficulties attended the operation rendering it protracted, dangerous and difficult. Yet I shall continue to advise the excision of the appendix in all such cases associated with a history of chronic relapsing inflammation, as has been detailed in these two cases just reported, as I think the operation in the interval less dangerous than the risk of perforation of the appendix and with the association of septic peritonitis.

